# Pharmaceutical services in primary health care: interfederative agreement in the development of pharmaceutical policies in the Brazilian Unified Health System (SUS)

**DOI:** 10.11606/S1518-8787.201705100supl2ap

**Published:** 2017-09-22

**Authors:** Karen Sarmento Costa, Noêmia Urruth Leão Tavares, José Miguel do Nascimento, Sotero Serrate Mengue, Juliana Álvares, Augusto Afonso Guerra, Francisco de Assis Acurcio, Orlando Mario Soeiro

**Affiliations:** I Núcleo de Estudos de Políticas Públicas. Universidade Estadual de Campinas. Campinas, SP, Brasil; II Programa de Pós-Graduação em Saúde Coletiva. Departamento de Saúde Coletiva. Faculdade de Ciências Médicas. Universidade Estadual de Campinas. Campinas, SP, Brasil; III Programa de Pós-Graduação em Epidemiologia. Faculdade de Medicina. Universidade Federal do Rio Grande do Sul. Porto Alegre, RS, Brasil; IV Departamento de Farmácia. Faculdade de Ciências da Saúde. Universidade de Brasília. Brasília, DF, Brasil; V Prefeitura Municipal de Florianópolis. Florianópolis, SC, Brasil; VI Programa de Pós-Graduação em Epidemiologia. Faculdade de Medicina. Universidade Federal do Rio Grande do Sul. Porto Alegre, RS, Brasil; VII Departamento de Farmácia Social. Faculdade de Farmácia. Universidade Federal de Minas Gerais. Belo Horizonte, MG, Brasil; VIII Faculdade de Ciências Farmacêuticas. Pontifícia Universidade Católica de Campinas. Campinas, SP, Brasil

The Brazilian Unified Health System (SUS) was established in 1990, in the institutional reorganization measures deriving from the promulgation of the Federal Constitution of 1988, for implementing the Health Policy recommended in the constitutional text^2^.

The implementation of a health system with such extent and comprehensiveness required promoting, preventive, and care policies in health and the institutionalization of new practices, arising from SUS’s principles and guidelines. In different countries, health systems present propositions of public policies, aimed at the population’s access to medications with proven quality, efficacy, and safety, besides the promotion of rational use of medicines, in systematic and constant interaction with the national policy^8^.

In Brazil, the *Política Nacional de Medicamentos* (National Policy of Medicines)^9^, the *Política Nacional de Assistência Farmacêutica* (National Policy of Pharmaceutical Services)^11^, and the *Política Nacional de Plantas Medicinais e Fitoterápicos* (National Policy of Medicinal Plants and Phytotherapy)^3^, included as part of the *Política Nacional de Saúde* (National Health Policy), also present the main purpose of achieving these propositions.

The creation of the Incentive to Basic Pharmaceutical Services represented a milestone in the organization of pharmaceutical services at this level of care. Criteria have been established for cities and states to qualify for receiving the incentive for basic pharmaceutical services, defining transfer values along with pharmaceutical technical guidelines required for such qualification^10^.

The decentralized model of pharmaceutical services in primary health care, with growing involvement of the local instance in the provision of pharmaceutical services to the population, represented new challenges in the management of health and pharmaceutical services for the population^19^ ^,^ ^23^.

In Brazil, the funding of the National Pharmaceutical Policies was traditionally tied to the definition and funding of outpatient medicines. In 2007, however, the financing and transfer of federal resources were regulated for health actions and services, as well as their monitoring and control, in the form of financing blocks, one of which on pharmaceutical services^12^ ^,^ ^23^.

This reorganization enabled the grouping of specific programs; helped the financial execution by SUS administrators; and provided greater clarity, transparency, and organization of the activities related to medicine management, aiming to ensure access for the population^22^.

The financing of pharmaceutical services according to this regulation is responsibility of the three spheres of SUS management (federal, state, and municipal), which agree on the standards for execution and the responsibilities within the Tripartite Intermanagement Committee (CIT). Federal resources are transferred to pharmaceutical services by three components: Basic, Strategic, and Specialized Component^12^ ^,^ ^23^.

Authors observe that the current model of funding for the Basic Component of Pharmaceutical Services uses, homogeneously in the country, a *per capita* value, and no other parameters that incorporate peculiarities of cities, regions, and states, as maintained until the last agreement, in 2013^16^.

A set of actions was made possible, aiming to support cities regarding the supply of medicines in health units, by participatory and decision-making support strategies in conjunction with municipal administrators^4^. Among these, authors highlight the purchases centralized by the Brazilian Ministry of Health and by some State Secretariats of Health^4^; the organization of municipal consortia for shared acquisition of medicines^1^ ^,^ ^7^; and the creation of the *Programa Farmácia Popular* (Popular Pharmacy Program), in two modes – the own pharmacies and *Aqui tem Farmácia Popular* (composed of accredited private pharmacies)^6^ ^,^ ^21^.

Also, in the provision of medicines, activities related to the selection of medicines are a relevant measure to consolidate Health Policies in developed and developing countries. In the Brazilian case, the ongoing review process of the *Relação Nacional de Medicamentos Essenciais* (Rename – National List of Essential Medicines) played a key role in the selection of medicines and improvement of pharmaceutical services to the user. This instrument has enabled the country to rely on lists of medicines, periodically and systematically updated, to guide states and cities especially in matters related to the process of selecting medicines to be offered to users. The process began in 1964 and resulted in 11 updates, until the 2014 version^18^.

On the other hand, the lack of nationwide data on pharmaceutical services in primary health care represented a gap in the management, evaluation, and redirection of Pharmaceutical Policies in SUS^5^.

To address part of this gap, different strategies have been developed and agreed between all three instances of SUS, as in the *Sistema Nacional de Gestão da Assistência Farmacêutica* (Hórus – National System of Pharmaceutical Services Management)^16^ and the creation of the *Base Nacional de Dados de Ações e Serviços da Assistência Farmacêutica* (National Database of Pharmaceutical Services and Actions)^15^. The definition of data related to the management of the Basic Component, the recognition of the autonomy of federated entities to use their own tools, and the possibility of interoperability between computer systems allow one to identify, in fact, how pharmaceutical services in primary health care are organized and offered to citizens^5^.

From 1998 to 2008, during which the first three National Pharmaceutical Policies were promulgated, the demands of health services correlated to the structuring of pharmaceutical services in SUS had been repressed. Thereby, the structuring of pharmaceutical services began late and in mismatch with the managing and logistic activities of the area^22^.

The Ordinance GM/MS 1,555/2013 was the first tripartite agreement of financial compensation resources from state and municipal secretariats that advanced in the institutionalization of resources for structuring pharmaceutical services, when agreed in the Bipartite Intermanagement Committees^16^.

The first federal action with resources, directly related to supporting improvements in the qualification of pharmaceutical services in the country, referred to the regulation and agreement of the *Programa Nacional de Qualificação da Assistência Farmacêutica* (QUALIFAR-SUS – National Program for the Qualification of Pharmaceutical Services). Its purpose is to favor the process of consolidation and improvement of activities of pharmaceutical services; promote the systemic integration in health actions and services; and seek continuous, full, safe, responsible, and humanized care. The Program is structured in four complementary and integrated axes: structure, information, education, and care^13^.

This program promoted the transfer of federal resources for structuring pharmacies in the primary health care, by the Structure Axis of QUALIFAR-SUS^20^, and the development of implementation models of pharmaceutical care services in the cities, encouraged by the Care Axis of the Program^17^.

The improvement of the process of interfederative agreements, especially regarding the executive responsibilities and the financing of pharmaceutical services in primary health care, seeks to strengthen the participation of the three levels of Government in the administration of the policy and in the intermanager interactions and to promote strategies favoring the consolidation of pharmaceutical services in SUS, throughout the country.

We also understand that the agreed strategies shared in the Intermanagement Committees have sought to gradually establish closer ties with the municipal instance, to enhance local actions.

However, the agreement in the formulation of policies and programs must be based on reliable and up-to-date information. Thus, it will enable administrators to precisely formulate strategies that recognize regional differences and overcome inequalities in the population access to medicines and pharmaceutical services.

Before the investments and interfederative strategies introduced so far, we must identify in what way pharmaceutical services are put into practice in primary health care. Considering this demand, the Brazilian Ministry of Health has formulated, funded, and coordinated, along with researchers from different universities in the country, a specific research – the *Pesquisa Nacional de Acesso, Utilização e Promoção do Uso Racional de Medicamentos* (PNAUM – National Survey on Access, Use and Promotion of Rational Use of Medicines). The goal of PNAUM was to evaluate the access, use, and rational use of medicines by the Brazilian population, besides evaluating public policies and their effectiveness in SUS primary health care^14^.

The Component Evaluation of Basic Pharmaceutical Services of PNAUM provides data for the definition of priority demands to pharmaceutical services in primary health care; enables a better reflection on the model of funding and organization of pharmaceutical services; and subsidizes the planning, monitoring, and evaluation of these services. In addition, it shows to society the importance and need for public investment in Public Pharmaceutical Policies in Brazil. With this, we expect the results presented and discussed in this supplement can improve the process of interfederative agreement.
